# Spatial Analysis of the 2017 Outbreak of Hemorrhagic Disease and Physiographic Region in the Eastern United States

**DOI:** 10.3390/v13040550

**Published:** 2021-03-25

**Authors:** Christine L. Casey, Stephen L. Rathbun, David E. Stallknecht, Mark G. Ruder

**Affiliations:** 1 Southeastern Cooperative Wildlife Disease Study, College of Veterinary Medicine, University of Georgia, 589 D.W. Brooks Dive, Athens, GA 30602, USA; christine.casey@ky.gov (C.L.C.); dstall@uga.edu (D.E.S.); 2 Kentucky Department of Fish and Wildlife Resources, 1 Sportsman’s Lane, Frankfort, KY 40601, USA; 3 Department of Epidemiology & Biostatistics, College of Public Health, University of Georgia, 101 Buck Road, B.S. Miller Hall, Athens, GA 30606, USA; rathbun@uga.edu

**Keywords:** Appalachian Plateau, bluetongue virus, epizootic hemorrhagic disease virus, hemorrhagic disease, spatial analysis, white-tailed deer

## Abstract

Hemorrhagic disease (HD) is considered one of the most significant infectious diseases of white-tailed deer in North America. Investigations into environmental conditions associated with outbreaks suggest drought conditions are strongly correlated with outbreaks in some regions of the United States. However, during 2017, an HD outbreak occurred in the Eastern United States which appeared to be associated with a specific physiographic region, the Appalachian Plateau, and not drought conditions. The objective of this study was to determine if reported HD in white-tailed deer in 2017 was correlated with physiographic region. There were 456 reports of HD from 1605 counties across 26 states and 12 physiographic regions. Of the 93 HD reports confirmed by virus isolation, 76.3% (71/93) were identified as EHDV-2 and 66.2% (47/71) were from the Appalachian Plateau. A report of HD was 4.4 times more likely to occur in the Appalachian Plateau than not in 2017. Autologistic regression models suggested a statistically significant spatial dependence. The underlying factors explaining this correlation are unknown, but may be related to a variety of host, vector, or environmental factors. This unique outbreak and its implications for HD epidemiology highlight the importance for increased surveillance and reporting efforts in the future.

## 1. Introduction

Hemorrhagic disease (HD) is considered one of the most significant infectious diseases of white-tailed deer (*Odocoileus virginianus*) in North America [1,2]. Two closely related viruses, epizootic hemorrhagic disease virus (EHDV) and bluetongue virus (BTV), belonging to the genus *Orbivirus,* are the causative agents of HD [2]. These viruses are transmitted by biting midges in the genus *Culicoides* [3]. In North America, only *C. sonorensis* and *C. insignis* have been confirmed as competent vectors for BTV and/or EHDV; however, there is evidence suggesting other *Culicoides* species may be important vectors [4,5,6]. In the United States, *C. insignis* is largely limited to Florida, although there is recent evidence of a range expansion within the Coastal Plain physiographic region to nearby states [7]. The geographic range of *C. sonorensis* spans much of the United States but in the eastern part of the country is typically only sporadically detected, although *C. sonorensis* has been recently identified in southern Ontario [6,8]. It is not clear if such range expansions are temporary or permanent, but regardless, the distribution of *Culicoides* spp. is typically not static.

Hemorrhagic disease primarily affects white-tailed deer and mule deer (*Odocoileus hemionus*), but domestic ruminants also can be infected with both BTV and EHDV [2]. In Europe, several BTV serotypes have emerged, some of which were associated with widespread disease in cattle and sheep [9]. Likewise, reports of disease in cattle associated with EHDV have become more common worldwide [6,10]. The epidemiology of HD is complex and clinical manifestations in wild ruminants are highly variable. There are many factors that contribute to this complexity including but not limited to: vector biology, population immunity, pathogen virulence, environmental conditions, and landscape features. In white-tailed deer, infection with EHDV and BTV can range from subclinical to acute mortality and this clinical spectrum appears to be spatially dependent on endemic or epidemic patterns and resulting herd immunity [11,12].

Dynamic interactions between factors contributing to the complex epidemiology of HD produce the classic cyclic pattern of HD outbreaks [6]. In the eastern United States, outbreaks typically occur in the late summer and early autumn and there is a strong latitudinal gradient to the observed patterns, with endemic cycles in parts of the Southeast transitioning to epidemic cycles moving north [6]. However, in recent years, a northern expansion of HD has been observed in the midwestern and northeastern United States, where outbreaks were historically rare [13]. In addition to an expanding northern range, several exotic serotypes of both virus serogroups have been introduced into the United States over the past twenty years [14], some of which have been associated with severe disease and mortality in white-tailed deer [6]. These changing patterns highlight the need to understand the mechanisms driving these changes in HD epidemiology.

In 2017, there was a large HD outbreak in the eastern United States. The majority of HD activity was associated with EHDV-2, although EHDV-6 was detected in multiple states for the first time. Additionally, there was a large outbreak of EHDV-2 which appeared to be associated with the Appalachian Plateau physiographic region. These noteworthy changes in the epidemiology of HD prompted an investigation into the relationship between HD outbreaks and physiographic regions. The objective of this study was to determine if the 2017 HD outbreak in white-tailed deer was correlated with a specific physiographic region, the Appalachian Plateau. We hypothesized that the outbreak was spatially dependent on physiographic region. To investigate this relationship, we conducted an analysis on HD county-level reports from the eastern United States.

## 2. Materials and Methods

The 2017 county-level reports of HD occurrence were obtained from an ongoing long-term survey conducted by the Southeastern Cooperative Wildlife Disease Study (SCWDS; University of Georgia) that is sent to all state wildlife agencies, and which has been previously described [1]. There are four criteria for HD reporting (1) sudden and unexplained high mortality during the late summer and early fall in deer; (2) diagnosis of HD based on necropsy findings; (3) viral isolation or molecular-based detection of EHDV or BTV; and/or (4) observation of hunter-killed deer that showed sloughing hooves, oral ulcers, or scars on the rumen lining. The first three criteria are associated with direct mortality, whereas criterion 4 is associated with chronic disease progression resulting in morbidity or indirect mortality. In addition to the annual survey, SCWDS annually performs diagnostic testing on tissue samples from dead wild ruminants suspected to have died from HD. Tissue samples are submitted by state wildlife agency personnel and samples are processed and tested as previously described [15].

The study area consisted of 1605 counties from 26 states in the eastern United States including: Alabama, Connecticut, Delaware, Florida, Georgia, Illinois, Indiana, Kentucky, Maine, Maryland, Massachusetts, Michigan, Mississippi, New Jersey, New Hampshire, New York, North Carolina, Ohio, Pennsylvania, Rhode Island, South Carolina, Tennessee, Vermont, Virginia, West Virginia, and Wisconsin. Physiographic regions were identified based on county-level data provided from a US Geological Survey Map [16]. Many counties covered more than one physiographic region within their boundaries. In these cases, counties were assigned based on the predominant physiographic type. Twelve major physiographic regions were represented. Physiographic regions describe the features and attributes of the Earth’s land surface and are influenced by geology and climate. Designations are based on distinct landscape types, terrain, land features, soils, rock type, and geologic history.

All summary statistics were computed using OpenEpi [17]. Odds ratios were computed as the odds of a positive report within a specific physiographic region over the odds of a positive report in the remaining region and were used to determine statistical significance for which physiographic regions were positive. Two regions were identified: the Appalachian Plateau and the Interior Low Plateau. These were used as independent variables in logistic regression models. A logistic regression model was used to assess whether the Appalachian Plateau physiographic region was a predictor of our dependent variable, a report of HD in a county during 2017. Three logistic regression models were tested for significance: general, specific, and expanded model. The “general model” was built under the assumption that a report based on one or more of the 4 criteria from the HD survey to indicate a positive county as the dependent variable and the Appalachian Plateau physiographic region as the independent variable. The “specific model” was constructed excluding criterion four from the definition of a positive report and the Appalachian Plateau physiographic region as the independent variable. The third “expanded model” used the specific model and added the Interior Low Plateau as an additional predictor. If the residual deviance of the logistic regression model exceeded the critical value, the model was not considered significant and not assessed for spatial dependency. The Akaike information criterion (AIC) was calculated in R and included for each of the above models. AIC was used to compare the relative fit of each logistic regression model. Centered autologistic models were created for both the specific and expanded models using maximum pseudolikelihood estimation [18]. All analyses were conducted in R (3.5.1) software package and logistic regression models were created using the package ‘stats’ and function ‘glm’ [19]. Graphics were produced using the following packages, “usmap” and “ggplot2”, in R (3.5.1). A neighborhood adjacency matrix was required for the centered autologistic models. First polygons of counties from US census data were obtained from shapefiles using the “tigris” package. Next, an adjacency matrix was constructed using ‘poly2nb’ function in the “spdep” package available in R (3.5.1). Using the “ngspatial” package, autologistic regression models were built by supplying the required arguments and setting method to pseudolikelihood estimation and bootstrap for confidence intervals [18]. Under the autologistic regression model, the log odds that HD is present is modeled as a linear function of the physiographic region and the number of adjacent counties in which HD is present.

## 3. Results

**Figure 1:**
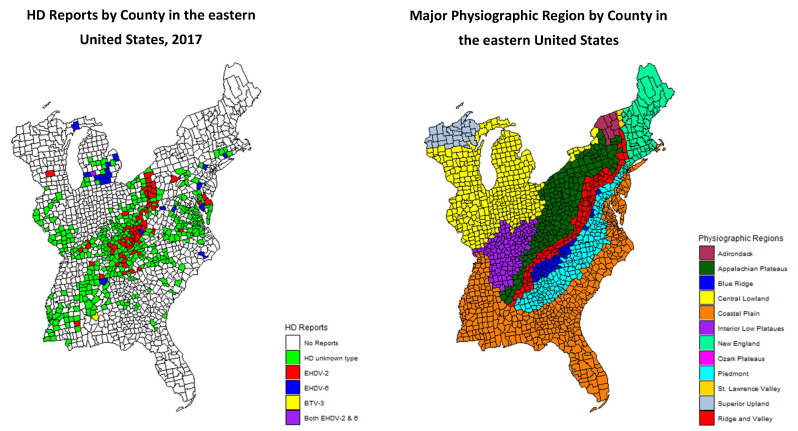
Map of hemorrhagic disease (HD) reports and virus isolation (epizootic hemorrhagic disease virus, EHDV, or bluetongue virus, BTV) results in 2017 by county in the eastern United States (left). Reports of HD that were not confirmed by virus isolation are green (*n* = 362), red counties were EHDV-2 positive (*n* = 71), blue counties were EHDV-6 positive (*n* = 20). There was one county positive for BTV-3 (yellow) and one county that was positive for both EHDV-2 and EHDV-6 (purple). The map on the right was created and represents the major physiographic region by county in the eastern United States using USGS data [16].

During 2017, one or more cases of HD were reported in 456 of 1605 counties, based on one or more of the four criteria (Figure 1). Odds ratios for reports of HD in the various physiographic regions are presented in Table 1. Of the twelve physiographic regions, three, Adirondack, St. Lawrence Valley, and Superior Upland, had no reports of HD in any county. One physiographic region, Ozark Plateau, only had one HD-positive county, and the odds ratio is not defined for that physiographic region. The odds ratios for a county to report positive for HD were significant for the Appalachian Plateau and the Interior Low Plateau. Of the 456 counties reported positive for HD, the statuses of 93 were confirmed by virus isolation (Table 1). Of these 93 counties with confirmed HD, 54% (50/93) were in the Appalachian Plateau region. Overall, the most common virus identified was EHDV-2 (76%; 71/93), of which the majority (66%; 47/71) were from the Appalachian Plateau. EHDV-6 was the second most common (23%; 21/93) virus isolated, and the majority (57%; 12/21) of EHDV-6 detections were in the Central Lowland physiographic region. Odds ratios were calculated for counties with HD reports based on the virus isolation data and physiographic region. The calculated odds ratio for EHDV-2 in the Appalachian Plateau was 8.1 compared to an odds ratio of 10.1 for EHDV-6 in the Central Lowland region. The Appalachian Plateau, as predictor variable, was significant in all three logistic regression models (Table 1). Additionally, in the expanded model the Interior Low Plateau was also statistically significant. The Akaike information criterion (AIC) was the lowest for the “expanded model.” The spatial dependence coefficients calculated in the autologistic models were statistically significant and the confidence intervals were narrow and did not contain zero for both models (Table 2). The reduced magnitudes of the coefficients for the physiographic regions under the autologistic regression model suggests that at least some of the effects of those regions may be explained by the spatial dependence in the data.

## 4. Discussion

The Appalachian Plateau was significantly correlated with reported HD in 2017. A report of HD was 4.4 times more likely to occur in the Appalachian Plateau than not in the Appalachian Plateau. Interestingly, statistical analysis of the data demonstrated that the Interior Low Plateau was also significantly correlated with reports of HD in 2017. A report of HD was three times more likely to occur in the Interior Low Plateau than not in the Interior Low Plateau. It is still unclear if there is a specific physiographic feature associated with these regions that is responsible for the increase in HD reports in 2017. While teasing out a specific landscape feature associated with these regions may be difficult, it is important to note that physiographic regions are controlled by geology and climate. Therefore, climatic features associated with specific regions could influence the prevalence of the vector. Additionally, one feature both regions have in common is that they are located on the western aspect of the Appalachian Mountains. *Culicoides* may be dispersed over long distances by wind, and wind direction has been implicated in long and short-range virus dispersal [20]. It is possible that the Appalachian Mountains provided a wind shield to funnel *Culicoides* from more southern enzootic areas throughout the Appalachian Plateau and adjacent Interior Low Plateau physiographic regions. However, while this hypothesis may help to explain the source and possible spatial distribution of EHDV-2 it is likely that other unknown factors affecting sustained transmission contributed to this outbreak. Further investigation is needed to fully identify the host of interacting risk factors that drive HD outbreaks, recognizing that there may be variation between outbreak years and locations.

One of the limitations of this study is reporting bias. It is possible because the high-profile nature and dramatic clinical manifestations of the disease that individual reports of HD beget additional reports due to increased public awareness and surveillance efforts. However, in this scenario, we suspect the observed correlation is not an artifact of this potential bias. Most of the reporting states agencies, including all of those surrounding the Appalachian Plateau are historically aware of HD and have reliably reported HD outbreaks when observed. In addition, there was widespread confirmation of these reports by virus isolation throughout the Appalachian Plateau (Figure 1).

When analyzing HD reporting data from the 456 affected counties by physiographic region and diagnostic virology data, the odds of EHDV-2 was 8.1 times more likely to occur in counties within the Appalachian Plateau than in other counties reporting HD. This supports the idea that the 2017 HD outbreak along the Appalachian Plateau was likely dominated by one serotype, EHDV-2. While there were three reports of EHDV-6 from the Appalachian Plateau, EHDV-6 was 10.1 times more likely to be isolated from counties reporting HD in the Central Lowland region. The odds ratio for isolating EHDV-2 from counties in the Interior Low Plateau reporting HD was 0.42. This is likely an underestimate due to the low number of HD reports confirmed by virus isolation from this region (7.9%, 6/76). The discrepancies between the odds ratios and the relationship between the physiographic region and serotype, highlights the importance for sustaining and enhancing efforts to confirm HD outbreaks by virus isolation. Based on our results, it is likely that independent outbreaks occurred in the Appalachian Plateau and Interior Low Plateau physiographic provinces.

A centered autologistic model was selected to minimize potential spatial confounding effects associated with the traditional autologistic model. The statistically significant spatial dependence coefficients support our hypothesis that HD reports associated with EHDV-2 during 2017 were correlated with the Appalachian Plateau. The “expanded” logistic model improved the fit compared with the “general” and “specific” models. However, the “expanded” autologistic model only marginally improved the accuracy of the model. The models in this analysis were simplistic and would likely benefit from including additional predictors like precipitation, average temperatures, drought, or wetland coverage. However, the intention of this analysis was to focus, specifically, on physiographic regions. These simple models demonstrate a spatial dependency and correlation between specific physiographic regions and HD reports in 2017. While not well investigated, physiographic regions may impact both the distribution of host and vector species, and subsequently their interactions, on the landscape. In the northern Great Plains, Schmidtmann and others (2011) showed that *Culicoides* spp. distribution may be defined in part by ecoregion and the presence or absence of glaciated soils in the region, suggesting physiographic region may be an important consideration in exploring the spatial distribution of HD [21]. The actual factors that enabled this outbreak to occur are still unknown, but it is important to emphasize that such factors may vary greatly between outbreaks. For example, while regional HD outbreaks have been associated with drought, such conditions were not present in outbreak areas during 2017. This unique outbreak and its implications for HD epidemiology underscore the importance for increased surveillance and reporting efforts in the future. Additionally, including virus isolation data and investigating potential predictors like host population structure, *Culicoides* population distribution and abundance, host density (including livestock), and climatic features will be important for enhancing our understanding and improving the accuracy of predictive models.

## Figures and Tables

**Table 1:** Odds ratios for reported and confirmed hemorrhagic disease (HD) in the eastern United States during 2017 based on physiographic regions. Epizootic hemorrhagic disease viruses and bluetongue viruses were identified on a subset of deer with suspected HD. There was a total of 456 counties that reported at least one or more cases of hemorrhagic disease.

Physiographic Region	Odds Ratio	95% Confidence Interval	Frequency of Counties w/Reported HD	Virus Detection Frequencies			
				EHDV-2	EHDV-6	BTV-3	EHDV-2 & -6
Adirondack	0	-	(0/7)	-	-	-	-
Appalachian Plateau	4.37	3.24, 5.89	57.8% (122/211)	47	3	0	0
Blue Ridge	1.85	1.0, 3.43	41.9% (18/43)	5	0	0	0
Central Lowland	0.43	0.31, 0.58	16.5% (58/352)	2	11	0	1
Coastal Plain	0.49	0.38, 0.64	18.8% (83/441)	4	2	1	0
Interior Low Plateau	3.04	2.15, 4.31	51.7% (76/145)	6	0	0	0
New England	0.21	0.07, 0.48	7.8% (5/64)	0	1	0	0
Piedmont	1	0.72, 1.39	28.4% (56/197)	1	1	0	0
St. Lawrence Valley	0	-	(0/4)	-	-	-	-
Superior Upland	0	-	(0/28)	-	-	-	-
Ridge and Valley	1.27	0.84, 1.9	33% (37/112)	6	2	0	0
*TOTAL*			28.4% (456/1605)	71	20	1	1

**Table 2:** Coefficients for logistic regression models for reported hemorrhagic disease activity in the eastern United States during 2017. The coefficients and confidence intervals for the logistic regression models on top. The coefficients and confidence intervals for the fitted centered autologistic “specific” and “expanded” models at the bottom. Confidence intervals are given in parentheses. The deviance test showed that while the specific and expanded models both adequately fit the data, the general model showed a significant lack of fit. The model with the lowest Akaike information criterion (AIC) value represents the model with the best fit.

**Logistic Regression Model**	**Appalachian Plateau**	**Interior Low Plateau**	**AIC**	**Residual Deviance**	**Critical Value (X^2^)**
General Model	1.45 (1.18, 1.73)	-	1814	1810	1697.3
Specific Model	1.59 (1.31, 1.87)	-	1688	1684	1697.3
Expanded Model	1.74 (1.45, 2.03)	1.59 (1.23, 1.96)	1618.5	1612.5	1696.2
**Autologistic Regression Model**	**Appalachian Plateau**	**Interior Low Plateau**	**Coefficient of Spatial Dependence**		
Specific Model	0.46 (0.02, 1.29)	-	0.91 (0.80, 1.04)		
Expanded Model	0.52 (0.08, 1.41)	0.60 (0.08, 2.14)	0.90 (0.79, 1.01)		

## Data Availability

The data presented in this study are available on request from the corresponding author. The data are not publicly available due to privacy restrictions.
